# An agent-based simulation model to study accountable care organizations

**DOI:** 10.1007/s10729-014-9279-x

**Published:** 2014-04-09

**Authors:** Pai Liu, Shinyi Wu

**Affiliations:** 1 Daniel J. Epstein Department of Industrial and Systems Engineering, University of Southern California, Los Angeles, CA USA; 2 Palo Alto Research Center, Palo Alto, CA USA; 3 School of Social Work, University of Southern California, Los Angeles, CA USA; 4 RAND Corporation, Santa Monica, CA USA; 5 School of Social Work and Daniel J. Epstein Department of Industrial and Systems Engineering, University of Southern California, Los Angeles, CA 90089-0411 USA

**Keywords:** Agent-based model, Health policy simulation, Accountable care organization, Congestive heart failure, Payment model

## Abstract

Creating accountable care organizations (ACOs) has been widely discussed as a strategy to control rapidly rising healthcare costs and improve quality of care; however, building an effective ACO is a complex process involving multiple stakeholders (payers, providers, patients) with their own interests. Also, implementation of an ACO is costly in terms of time and money. Immature design could cause safety hazards. Therefore, there is a need for analytical model-based decision-support tools that can predict the outcomes of different strategies to facilitate ACO design and implementation. In this study, an agent-based simulation model was developed to study ACOs that considers payers, healthcare providers, and patients as agents under the shared saving payment model of care for congestive heart failure (CHF), one of the most expensive causes of sometimes preventable hospitalizations. The agent-based simulation model has identified the critical determinants for the payment model design that can motivate provider behavior changes to achieve maximum financial and quality outcomes of an ACO. The results show nonlinear provider behavior change patterns corresponding to changes in payment model designs. The outcomes vary by providers with different quality or financial priorities, and are most sensitive to the cost-effectiveness of CHF interventions that an ACO implements. This study demonstrates an increasingly important method to construct a healthcare system analytics model that can help inform health policy and healthcare management decisions. The study also points out that the likely success of an ACO is interdependent with payment model design, provider characteristics, and cost and effectiveness of healthcare interventions.

## Introduction

The current health system in the United States lacks mechanisms and incentives for providers to control rapidly rising healthcare costs and improve quality of care [1]. Hence, an idea was proposed in 2007 of an accountable care organization (ACO), a group of providers who accept greater accountability for total costs and quality of care [2, 3]; this has been widely discussed since it was outlined in the Patient Protection and Affordable Care Act [4] in 2010. The Centers for Medicare & Medicaid Services (CMS) have established a Medicare Shared Savings Program to create ACOs implementing shared saving payment models [5]. In addition, many private insurers and healthcare organizations have launched or will launch their ACO demonstrations in the near future [6].

However, an ACO could implement a broad range of delivery and payment models (capitation, bundled payment, shared saving) [7]; it is unclear how ACOs should be structured and implemented [8] to deliver high-quality care and control spending. Meanwhile, an ACO demonstration is costly in terms of time and money and could cause safety hazards because of immature design. Therefore, the design and implementation of ACOs are complex and risky, and there is a need for predictive analytics tools that can predict future outcomes of different ACO models and generate recommendations based on goals set by policymakers.

Industrial and Systems Engineering (ISE) methods have the potential to address this need. However, traditional ISE approaches are facing challenges in modeling some important features of an ACO, which is composed of multiple interacting individual stakeholders from multiple sectors–including payers, providers, and patients–who are working to maximize their own interests. Consequently, the Agency for Healthcare Research & Quality (AHRQ) has called for methodologies that can incorporate different objectives and behaviors of multiple interacting stakeholders from different sectors into the model and make optimal recommendations for the overall system [9]; AHRQ has identified the methodologies as a critical area of ISE research in healthcare.

The main contribution of this article is that an agent-based model was developed to address the needs of predictive analytics for ACOs from a cross-sectoral point of view. By combining different agents’ varying goals and behaviors from their vantage points in a system model, the analysis reveals new, integrated insights to help inform health policy and healthcare management decisions. A simulation study that analyzes ACOs aiming to deliver better care for congestive heart failure (CHF) was also developed to demonstrate the use of the agent-based model. CHF was selected in the model because it is the leading cause for hospitalization and high healthcare costs for elders [10]. In addition, multiple meta-analyses of clinical trials have shown that interventions of disease management programs could reduce CHF readmission rates and improve other health outcomes, including survival rates and quality of life [11, 12]. Therefore, payers and ACOs have the potential to create incentives and mechanisms to facilitate the adoption and implementation of this evidence-based care. The goal of the agent-based simulation model is not to substitute an ACO pilot program but to facilitate ACO design and implementation processes by providing analytic supports for decision-makers to make informed decisions.

A shared saving payment model for the ACOs was examined in the agent-based model because it is a popular payment model used in the CMS Shared Savings Program [5] and many other current and planned ACO demonstrations. Furthermore, it serves as an important bridge between the traditional fee-for-service model and more fundamental risk-sharing payment models, such as episode-based payment. Under shared saving payment, if a group of providers can control or reduce the total healthcare costs of a patient population while meeting quality measures, a portion of the saving will be awarded to the providers.

In this paper, Section 2 describes the agent-based model developed and a simulation study to demonstrate the model. Section 3 presents the results of the simulation study. A baseline analysis was conducted to compare the performance of an ACO health network and a controlled health network. In addition, multiple scenario analyses were conducted to examine the impacts of payment model designs and provider characteristics on the outcome measures.

## Method

In this chapter, the agent-based model developed to provide predictive analytics for ACOs is described, followed by the modeling settings of the simulation study for CHF patients. Section 2.1 briefly introduces the methodology of agent-based modeling and its advantages to model ACOs. Section 2.2 describes the framework of the agent-based model and the designs of each type of agent, including agent’s objectives, characteristics, behaviors, and how an agent interacts with other agents. Section 2.3 provides a gain and cost analysis from different agents’ perspectives in order to model agent decision-making. Section 2.4 discusses the configurations of the simulation run to demonstrate the model.

### Agent-based modeling

Agent-based modeling (ABM) can be viewed as a mindset rather than a specific modeling technique. ABM describes a complex system from the bottom-up perspective and models it as a collection of multiple autonomous agents who have their own objectives, behaviors, and interactions with other agents and the environment. As in the real world, global behaviors and trends emerge from the behavior of and interactions between individual agents [13, 14].

ABM can model individual behaviors of each stakeholder in an ACO and their dynamic interactions. This is a limitation and difficulty of traditional equation-based or discrete event modeling approaches. Because providing coordinated care is an essential part of an ACO, the ability to model agent collaborations and communications is a required function for an ACO model. The structure of ABM makes it easy to define dynamic agent interactions through functions such as message passing and agent networks.

Because ABM does not require predefined aggregate level flow and structure (as in System Dynamics), it is more flexible to expand through adding agent behaviors, interaction rules, and learning capacity. In addition, working as a platform, ABM can incorporate other modeling techniques. For example, it can apply artificial intelligence to model agent behavior changes and can incorporate stochastic process models to simulate agent internal state transitions. Furthermore, because ABM models the system from the individual agent level, it can take advantage of the increasing amount of individual level data.

### Model description

**Fig. 1:**
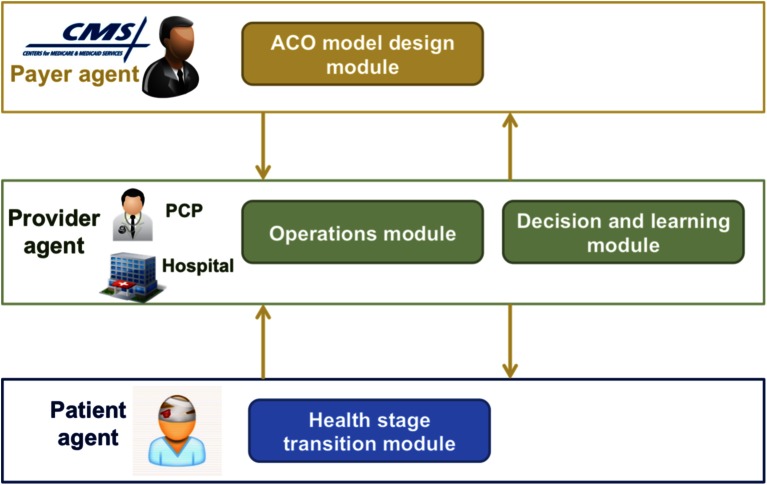
Agent-based model structure

As shown in Fig. 1, the agent-based model includes three types of agents who are the key stakeholders of an ACO demonstration: the patient agent, the provider agent, and the payer agent. Two classes of provider agents were defined because they are the main providers involved in CHF care: the hospital agent and primary care physician (PCP) agent. Under each agent, there are modules, each of which represents a set of related functions for the agent.

#### Patient agent

A patient agent denotes a Medicare patient whose age is 65 or older. Each patient agent is characterized by four demographic variables (age, race, gender, and income) and three health condition variables (diabetes, hypertension, and CHF). These variables were the key factors that have impacts on CHF disease progression and healthcare utilization [15–18].

Patient agent characteristics were estimated from the National Health and Nutrition Examination Survey (NHANES) of 1999–2010. Because the variables of a patient agent are correlated, the joint distribution of these variables was estimated on the basis of a conditional probability model:

$$ \begin{array}{c}\hfill P\left(\mathrm{CHF}\kern0.5em \mathrm{and}\kern0.5em \mathrm{Hypertension}\kern0.5em \mathrm{and}\kern0.5em \mathrm{Diabetes}\kern0.5em \mathrm{and}\kern0.5em \mathrm{Demographics}\right)=P\left(\mathrm{CHF}\Big|\mathrm{Hypertension}\ \mathrm{and}\right.\hfill \\ {}\hfill \left.\mathrm{Diabetes}\kern0.5em \mathrm{and}\kern0.5em \mathrm{Demographics}\right)\ast P\left(\mathrm{Hypertension}\Big|\mathrm{Diabetes}\ \mathrm{and}\right.\hfill \\ {}\hfill \left.\mathrm{Demographics}\right)\ast P\left(\mathrm{Diabetes}\Big|\mathrm{Demographics}\right)\ast P\left(\mathrm{Demographics}\right)\hfill \end{array} $$

**Fig. 2:**
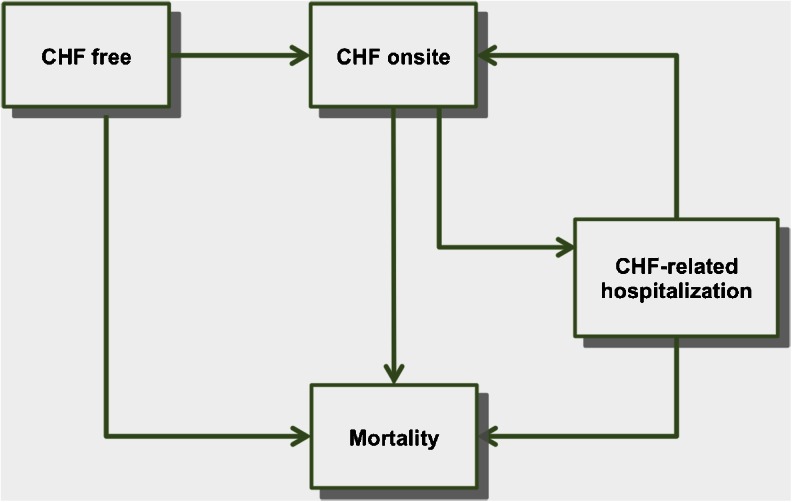
Patient agent transition model

The patient agent’s CHF disease progression and healthcare resource utilization are modeled in the patient agent state transition module shown in Fig. 2. A patient agent starts in either the CHF free state or CHF onsite state, depending on whether the agent has CHF when he/she was generated. In the CHF free state, a patient agent is exposed to the risk of developing CHF in either the outpatient setting (moving to the CHF onsite state) or the inpatient setting (moving to the CHF-related hospitalization state). A patient agent in the CHF onsite state is at risk of CHF-related hospitalization (moving to the CHF-related hospitalization state). Patient agents in any of the above states also have the possibility of death (moving to the mortality state, in which case the patient agent will be deleted from the simulation).

The CHF-related hospitalization was defined as a hospitalization in which CHF is listed in any of its first seven diagnoses. Because this model targets CHF care, non–CHF-related hospitalizations were not included. In addition, patient agents who have not been diagnosed with diabetes or hypertension are exposed to the risks of developing diabetes or hypertension. Because CHF, diabetes, and hypertension are incurable chronic diseases, once a patient agent develops any of these diseases, he/she will have it for the rest of the simulation.

There is a wide range of cycle time in the CHF modeling literature depending on the measurement needs of the studies [19–21]. The cycle time in the patient state transition module was set to half a month (15 days) in order to catch hospital readmissions within 30 days since previous discharge. At each cycle, a patient agent will move to other states or remain at the current state according to state transition probabilities, which are determined by the patient agent’s characteristic variables and are adjusted if the patient agent receives the CHF intervention care. The intervention here refers to the comprehensive discharge planning with post-discharge follow-up. More details about it can be found in Phillips et al. [12].

The impacts of these risk factors on the state transition probabilities are derived from the literature and public health surveys and statistics. First, the CHF incidence rate is risk-adjusted by the patient’s age group [22], diabetes [23, 24], and hypertension [16, 18, 24]. With regard to the mortality rate of CHF patient agents, Curtis et al. [22] showed there is a significant difference in mortality rate depending on whether the patients were first diagnosed with CHF in an inpatient setting or an outpatient setting. Thus, the diagnosis source was added as a risk factor for mortality, and two separate survival curves were constructed from data reported by Curtis et al. [22] (shown in Table 1) for each diagnosis source.

**Table 1:** CHF survival curve

Time since onset of CHF	Survival proportions	
	Outpatient diagnosis	Inpatient diagnosis
0	1.00	1.00
30 days	0.98	0.84
1 years	0.87	0.66
5 years	0.49	0.33

For non-CHF patient agents, the mortality rate was estimated from the U.S. life tables of 2006 [25]. The CHF-related hospitalization rate was adjusted by age group based on the estimations from Fang et al. [26] and Chen et al. [27] shown in Table 2. Diabetes incidence rates were developed based on McBean et al. [28], and hypertension incidence rates were calculated from Dannenberg et al. [29] and Dischinger et al. [30] as a function of patient’s race.

**Table 2:** Transition probability from the CHF-diagnosed state to CHF-related hospitalization

Age (years)	Transition probability
65–74 (age group 1–2)	0.01663
75–84 (age group 3–4)	0.02360
> =85 (age group 5)	0.03489

A patient agent generates inpatient care utilization by moving into the CHF-related hospitalization state. For outpatient care utilization, each patient agent’s regular outpatient visits were modeled as a Poisson process because it is a common approach to modeling patient arrivals [31–33]. The average number of regular outpatient visits estimated from the literature ranges from eight to ten visits per year [21, 34, 35]. Hence, the parameter of the Poisson process is set to nine visits per year. In addition to regular visits, a follow-up visit after hospital discharge will be scheduled if a patient agent receives the intervention.

#### Provider agent

The model includes two types of provider agents: the hospital agent and PCP agent; each represents a hospital or a PCP clinic, respectively. The goal of a provider agent is to maximize the utility of the hospital or clinic by deciding whether to conduct the CHF intervention.

The CHF intervention has been demonstrated by a number of clinic trials and meta-analysis studies as able to reduce hospital readmission and mortality rate for CHF patients [11, 12]. The effects of the intervention are modeled by reduction in transition probabilities to the CHF-related hospitalization state and mortality state. The risk reduction was derived from a meta-analysis of CHF interventions [12]. The average risk reduction is 20 % for CHF hospitalization rate and 13 % for CHF mortality rate.

Because most published CHF intervention studies involve collaboration of hospitals and PCPs [11, 12], the full effects of the intervention, it was assumed, could only be achieved when both the hospital where a patient was admitted and the patient’s PCP conducted the intervention. If only one party was conducting the intervention without collaboration with the other party, the intervention could only achieve partial effect.

**Fig. 3:**
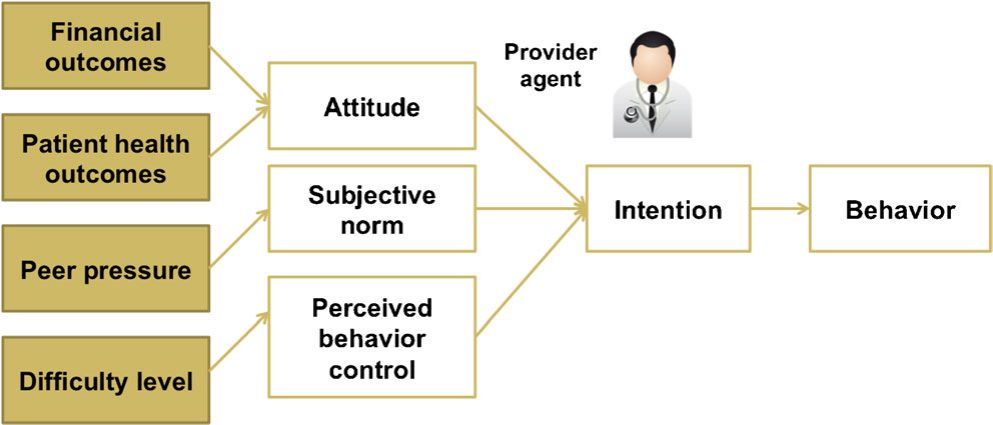
Application of the theory of planned behavior

The well-established psychological theory, the Theory of Planned Behavior (TPB) [36], was applied as the framework to model the provider agent decision-making. As shown in Fig. 3, TPB suggests that a person’s actual behavior is determined by his/her intention to perform the behavior, and the intention is influenced by three predicting factors: attitude, subjective norm, and perceived behavior control (PBC).

Attitude in TPB is a person’s belief about the consequences of a particular behavior. In the model, the provider agent’s attitude toward the intervention is influenced by his/her financial return as well as his/her client’s health outcomes measured by the CHF hospitalization rate and mortality rate. Hence, attitude A of a provider agent is formalized as

$$ {A}_{j+1}^k={\beta}_1\cdot {U}_p\left(E{P}_{j+1}^k\right)+{\beta}_2\cdot {U}_q\left(E{Q}_{j+1}^k\right), $$

where *EP*_*j* + 1_^*k*^ and *EQ*_*j* + 1_^*k*^ are the expected profit and expected quality performance, respectively, if behavior k will be performed in year j + 1. U is a function that transforms the expected profit or the expected quality performance into a utility value from 0 to 1. *β* is a weight parameter that can be configured by users.

Subjective norm is a person’s perception of whether people important to him/her believe he/she should perform a behavior. The provider agent’s subjective norm was defined in terms of peer pressure, which is a provider agent’s perception of other provider agents’ attitudes toward the intervention. The perception of other agents’ attitudes is modeled through agent communication. Based on his/her current attitude toward the CHF intervention, a provider agent will send a supportive message either for the behavior of implementing the intervention or for the opposite behavior of not implementing the intervention to a random agent within his/her network. A provider agent can then estimate other provider agents’ attitudes based on the number of supportive messages received for each behavior. Hence, the subjective norm is expressed by

$$ S{N}_{j+1}^k={U}_{sn}\left(n{m}_j^k\right), $$

where *nm*_*j*_^*k*^ is the number of supportive messages received for behavior k during year j, and *U*_*sn*_ is a utility function that transfers the number of messages into a utility value ranging from 0 to 1.

The third predicting factor, PBC, is a person’s perceived ease or difficulty of performing a particular behavior. As the CHF intervention is a collaborative behavior between hospitals and PCPs, the effects of the intervention are compromised if only one side of the agents conduct the intervention. Therefore, an agent’s perceived ease or difficulty at successfully performing the intervention is influenced by other agents’ behaviors. Hence, the PBC of a provider agent is defined as the perception of the percentage of other agents who are actually conducting the intervention. The process was modeled through agent interactions between hospital agents and PCP agents. An interaction happens when a patient agent is discharged and its hospital agent and PCP agent communicate to update the patient agent’s information and simultaneously to perceive if the other agent is conducting the intervention.

The PBC for the behavior of conducting the intervention (*k* = 1) in year *j + 1* is formalized as

$$ PB{C}_{j+1}^1=\raisebox{1ex}{$n{i}_j$}\!\left/ \!\raisebox{-1ex}{$t{i}_j$}\right., $$

where *ni*_*j*_ is the number of interactions in which the other agent is conducting intervention responses and *ti*_*j*_ is the total number of interactions. Because an agent does not need any collaboration from other agents to not conduct the intervention (*k* = 0), *PBC*_*j* + 1_^0^ was set to 1.

After the intention is formed, a softmax function can be used to calculate the probability that a provider agent will choose to conduct the intervention:

$$ {P}_{j+1}^1=\frac{ \exp \left({I}_{j+1}^1/\tau \right)}{ \exp \left({I}_{j+1}^0/\tau \right)+ \exp \left({I}_{j+1}^1/\tau \right)}. $$

The parameter *τ* is used to adjust the irrational level of decision-makers. In the demonstrating simulation study, rational decision-making was assumed, so the behavior with higher intention will be performed. The users of the agent-based model can set the parameters to reasonable values for their specific context or learn them empirically.

Last, to examine the impacts of provider characteristics, three hypothetical types of provider agents were defined in the demonstrating simulation study by varying the weights of *β* = [*β*_1_, *β*_2_]. The three types of provider agents are profit-oriented with *β* = [0.8, 0.2], quality-oriented with *β* = [0.2, 0.8], and neutral with *β* = [0.5, 0.5]. Scenario analyses with different mixes of these three types of provider agents were conducted and are described in Section 3.

#### Payer agent

The payer agent hypothetically represents CMS in the model because it is the primary payer for CHF healthcare services. (Note. In the text below, all references to CMS are hypothetical. The study does not reflect the view of the CMS, rather solely the views of the authors.) The CMS will collect cost and quality data, as well as calculate and distribute the shared saving. To calculate the shared saving, two provider agent networks were included: the ACO network and controlled network, each of which serves a patient population with identical patient characteristics. At the end of each year or decision cycle, the total CHF-related healthcare costs per CHF patient will be calculated for each network. The cost difference between these two networks is the saving per CHF patient from the payer’s perspective.

One key parameter of the shared saving payment model is shared saving rate (SSR), which defines the percentage of the total saving that will be awarded to providers. The shared saving per CHF patient is calculated by multiplying the saving per CHF patient with SSR, and the total shared saving is determined by multiplying the shared saving per CHF patient with the number of CHF patients that the ACO network serves.

The total shared saving will then be distributed among hospital agents and PCP agents in the ACO network. The distribution of the shared saving is defined by another key parameter: sharing rate to hospital (SRH), which is the percentage of the total shared saving distributed to hospital agents. Within each type of provider agent, the shared saving was assumed to be distributed equally. If the cost per CHF patient of the ACO network is higher than that of the controlled network, there will be no saving.

### Healthcare costs

The cost analysis was conducted from different agents’ perspectives. The cost for the payer agent representing CMS is the reimbursement paid to providers for CHF-related services plus the shared saving. For CHF-related hospitalization, the AHRQ’s estimation [37] is used for costs of hospital service, and the physician inpatient cost is estimated to be 18 % of the reimbursement for hospital service [21, 38]. The reimbursement for outpatient visits is determined by the CMS Physician Fee Schedule [39].

For a hospital agent, negative margins were reported for hospital services of Medicare patients [40, 41]. A −8.4 margin for CHF inpatient services was estimated based on Medicare’s reported reimbursement [42] and hospital operating costs [43, 44]. For CHF intervention cost, a meta-analysis of CHF interventions [12] showed that the pooled intervention cost was $108 per patient per month for a usually 6-month-long intervention. The intervention effects were assumed to last for 1 year, which gives us an average cost of $54 per patient per month.

A PCP agent’s operating cost was estimated to be 60 % of its revenue [45]. The intervention cost from a PCP agent’s perspective was defined as the opportunity cost of communicating with CHF care managers. If the time used for communication was 15 min per patient per year, it could be otherwise used for a standard outpatient visit reimbursed at $69 [39]. The healthcare costs are summarized in Table 3, and all values are converted into USD (2011) using the U.S. Consumer Price Index (CPI).

**Table 3:** Healthcare costs

Service	Amount paid by agent, USD (2011)		
	Payer agent	Hospital agent	PCP agent
CHF hospital cost	−14,822	−1,186	
Inpatient physician fee	−2,668		
Outpatient visit	−85		34
Intervention cost per patient per year		−648	−69

### Model implementation

The demonstrating simulation study was implemented using AnyLogic version 6.6 (XJ Technologies, Chicago, IL). In the study, the simulation time was set at 5 simulation years, which is reasonable to test the effects of an ACO demonstration.

The model generates one singlepayer agent hypothetically representing CMS and two networks of provider agents (one ACO network and one controlled network). Both the provider networks are composed of three hospital agents and 15 PCP agents. The ACO network is reimbursed under the shared saving model, and the controlled network is reimbursed under the traditional Medicare fee-for-service (FFS) model. The simulation model generated 10,000 patient agents and randomly assigned them to the ACO networks or the controlled network. For each patient agent, the values of its characteristic variables were assigned on the basis of the joint distribution estimated.

At the end of each simulation year, the payer agent will collect the performance measures for both provider networks, including the payment per CHF patient, the hospital admission rate, and the CHF patient mortality rate. Using those data, the payer agent will calculate and distribute the shared saving to the provider agents in the ACO network. Then, the ACO provider agents will go through the decision-making process to determine if they will conduct the intervention in the next simulation year.

The outputs of each simulation run are the average saving to the payer agent per CHF patient per year, average annual CHF-related admission rate, and CHF patient mortality rate for each provider agent network over 5 simulation years. For each setting of the model input parameters, 500 simulation runs are performed to calculate the mean and 95 % level confidence interval.

## Simulation results, model validation, and scenario analyses

A baseline analysis was conducted to compare the performance of an ACO health network and a controlled health network. Results below show the baseline model (Section 3.1) and its validation (Section 3.2). Section 3.3 describes the multiple scenario analyses conducted to examine the impacts of payment model designs and provider characteristics on the outcome measures. To examine the influence of provider types on the model outputs, three scenarios were created. The setting of the provider agent type in scenario one is the same with that in the baseline model. In scenario two, all the provider agents are profit-oriented, and in scenario three all the provider agents are quality-oriented. In each scenario, two key parameters of the shared saving payment model, i.e., SSR and SRH, were systematically varied to determine an optimal payment model design. The simulation was rerun for each combination of the values of SSR and SRH in each scenario. A sensitivity analysis was conducted to examine how the uncertainty in other key model inputs influenced the model outputs (Section 3.4).

### Baseline model

In the baseline model, the three hospital agents in the ACO network were a profit-oriented agent, a quality-oriented agent, and a neutral agent. A PCP agent in the ACO network had equal probability of being assigned as one of the three types. The key payment model parameters, SSR and SRH, were set at 0.5 and 0.7, respectively, which means half of the saving was shared with provider agents and 70 % of the shared saving was distributed to hospital agents.

The ACO network performed better in the CHF-related hospitalization rate and annual mortality rate of CHF patients owing to the implementation of evidence-based care. The annual CHF-related hospitalization rate was 63.94 % (95 % CI: 63.74–64.14 %) for the ACO network and 72.46 % (95 % CI: 71.78–73.14 %) for the controlled network. The annual mortality rate of CHF patients was 18.41 % (95 % CI: 18.33–18.49 %) for the ACO network and 19.72 % (95 % CI: 19.64–19.80 %) for the controlled network. From the payer’s or the CMS’s perspective, the average saving after distribution of the shared saving was $765 (95 % CI: $740–790) per CHF patient per year, or a 5.65 % saving compared with the $13,550 (95 % CI: $13,520–13,580) payment per CHF patient in the controlled network. The saving in the ACO network was mainly caused by the reduction in the hospitalization rate.

### Model validation

Because the controlled network provides usual care and does not conduct intervention, its CHF-related hospitalization rate and mortality rate were used to validate the model with the value reported in literature. The mortality rate for CHF patients reported in clinical trials ranges from 15 to 28 % [46–48]. Note that their sample sizes are usually small, and this could cause larger variation in their estimations. Hence, the mortality rate in the model (19.72 %) is comparable to and within the range of the mortality rates reported in the literature.

Derived from the results of Chen et al. [27] and Fang et al. [26], the CHF-related hospitalization rate was estimated around 68.6 %. In addition, the SOLVD study [49], a large CHF trial whose estimates have been used by many CHF simulation studies, reported an average annual hospitalization rate of 72.9 %. The CHF-related hospitalization rate in the model (72.46 %) is therefore comparable to that reported in the literature.

### Scenarios analyses

To examine the influence of provider types on the model outputs, three scenarios were created. The setting of the provider agent type in scenario one is the same as that in the baseline model. In scenario two, all the provider agents are profit-oriented, and in scenario three, all the provider agents are quality-oriented.

**Fig. 4:**
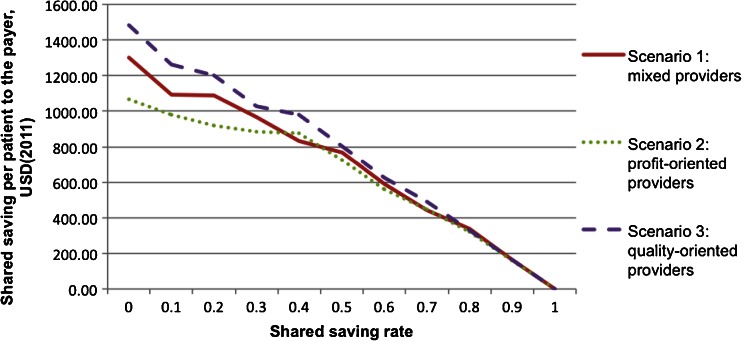
Shared saving to the payer by shared saving rate

**Fig. 5:**
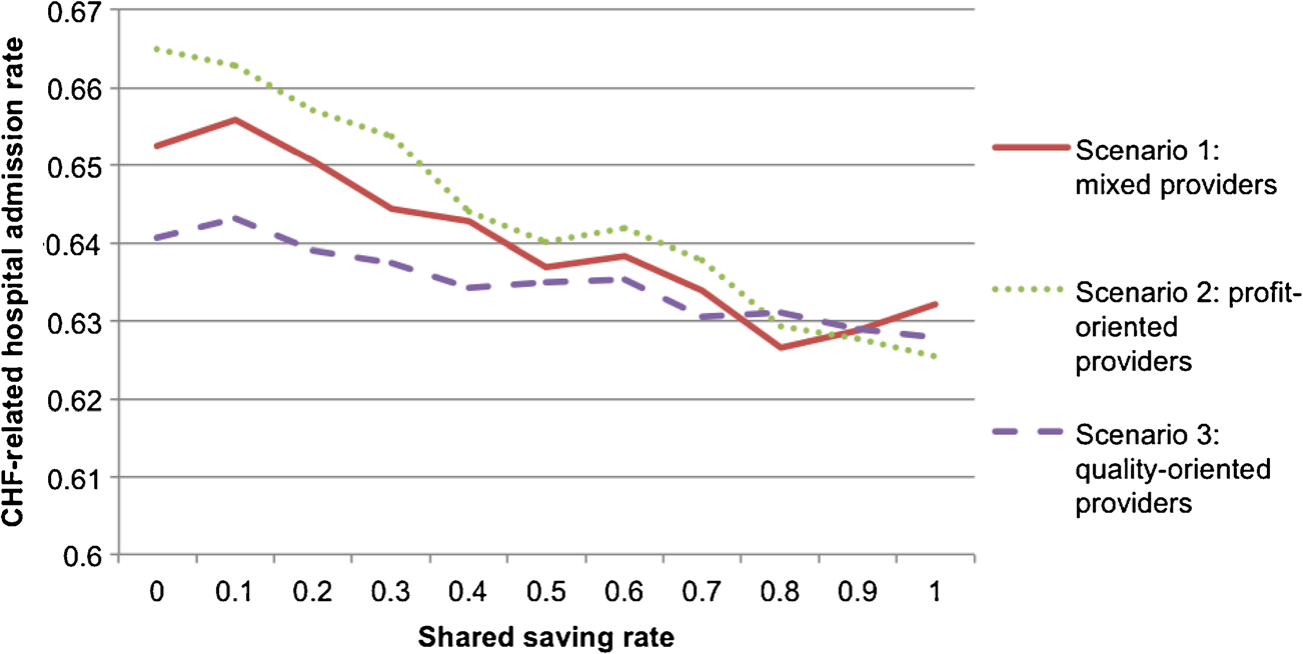
CHF-related hospitalization by shared saving rate

**Fig. 6:**
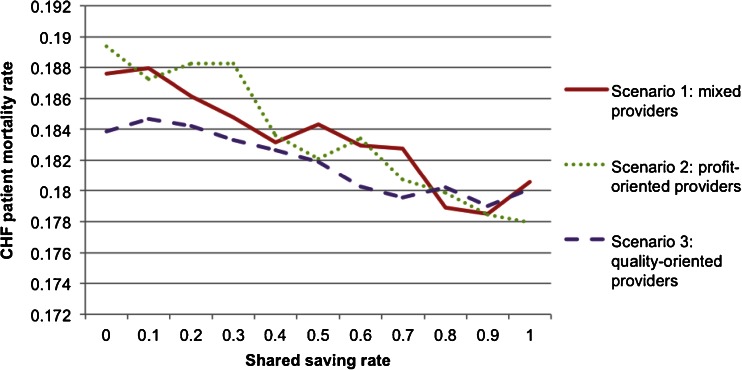
CHF patient mortality rate by shared saving rate

**Fig. 7:**
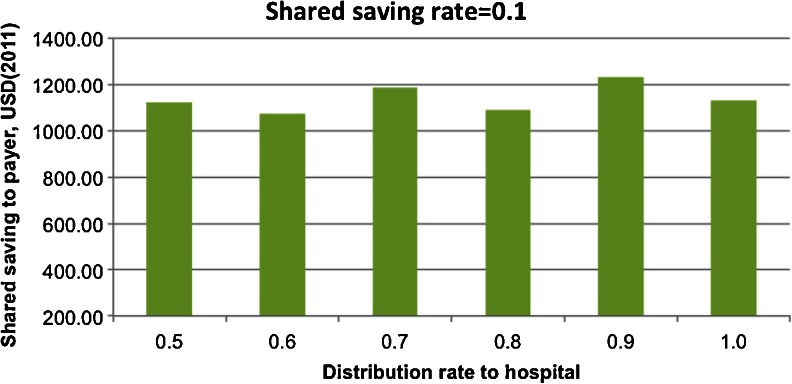
Shared saving to payer by sharing rate to hospital with the shared saving rate = 0.1

**Fig. 8:**
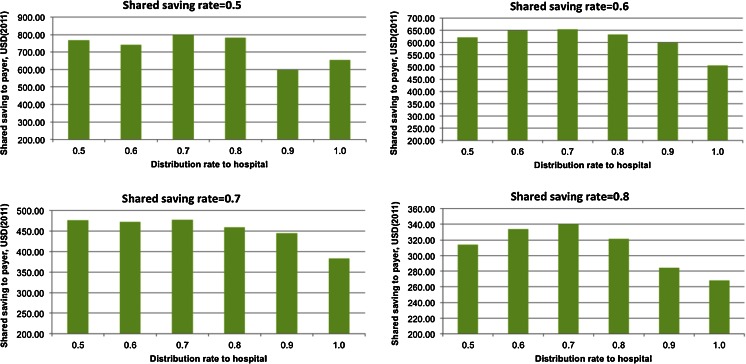
Shared saving to payer by sharing rate to hospital with shared saving rate from 0.5 to 0.8

**Fig. 9:**
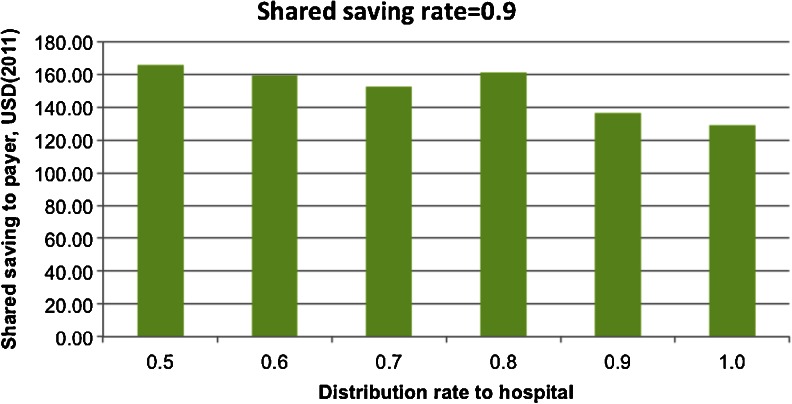
Shared saving to payer by sharing rate to hospital with the shared saving rate = 0.9

In each scenario, two key parameters of the shared saving payment model were systematically varied to determine an optimal payment (model design: SSR and SRH). The simulation was rerun for each combination of the values of SSR and SRH in each scenario. The results of the multiple runs were analyzed and used to generate Figs. 4, 5, 6, 7, 8, and 9.

Figure 4 shows that the shared saving to payer (SSP) decreases as SSR increases in all three scenarios. When the payer agent increases the SSR, the resulting additional financial incentives motivate more provider agents to conduct the intervention, causing a reduction in annual CHF-related hospital admission rate (shown in Fig. 5) and in annual CHF patient mortality rate (shown in Fig. 6). The reduction in the CHF-related hospital admission rate then generates more total saving in the system; however, the SSP actually decreases because more saving is shared with the provider agents. Therefore, the payer agent is faced with a trade-off between financial return and quality of care when determining the value of SSR.

This scenario analysis also shows how provider agents in different scenarios respond to changes in SSR. As shown in Fig. 4, given the same SSR, scenario three (all the provider agents are quality-oriented) always yielded higher financial return to the payer agent, followed in order by scenario one and scenario two. The profit-oriented provider agents are most sensitive to the changes in SSR.

The effects of varying SRH given a certain SSR were further examined. Analyzing the payer’s financial return (i.e., SSP by SRH in scenario one), different patterns were observed when the SSR was set at different values. When the SSR was low, there was no clear relationship between SSP and SRH (shown in Fig. 7 when the SSR was set at 0.1). Even if all the saving was distributed to hospital agents (when SRH is 1), it was not enough to cover the intervention cost for the hospital agents and motivate their behavior changes.

When SSR was set to 0.5–0.8, a pattern emerged with a concave shape, whereby the maximal financial return is achieved when the SRH is 0.7 (shown in Fig. 8). Because the effects of the intervention can be fully achieved only when both the hospital agents and PCP agents are conducting it, the 0.7 value of SRH seems to be a good balance point that can motivate both the hospital and PCP agents to conduct the intervention and thus achieve a maximal financial return for the payer agent.

When SSR was high (0.9), Fig. 9 shows a relatively flat trend of SSR when SRH was around 0.5–0.7, and when the SRH was greater than 0.7, SSR decreased as SRH increased. Because the hospital agent had enough financial incentives to conduct the intervention, a clear hospital agent behavior switch was not observed as SRH increased from 0.5 to 0.7. Similar patterns have also been observed in the other two scenarios.

### Sensitivity analysis

After the scenario analyses were conducted on the shared saving payment model and provider types, a sensitivity analysis was conducted to examine how the uncertainty in other key model inputs influenced the model outputs (using SSP). In general, for each testing parameter, a higher value (increased by 20 %) and a lower value (decreased by 20 %) were tested.

**Fig. 10:**
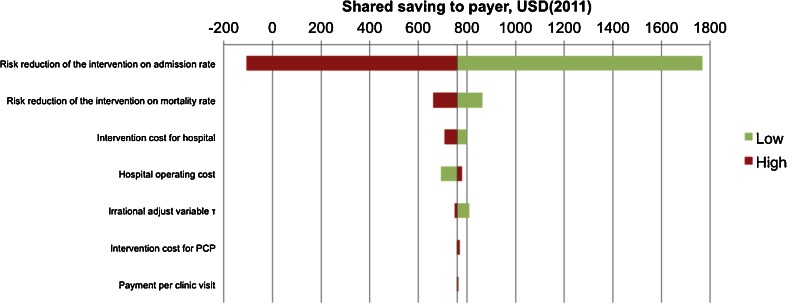
Sensitivity analysis to identify influence of model inputs uncertainty on the shared saving to payer output

From the results (shown in Fig. 10), SSP was most sensitive to the effects of the intervention on hospital admission rates. This was expected because the saving was mainly generated by the reduction in hospitalization. Next, the effects of the intervention on CHF patient mortality rate were discussed. This is important because the quality outcomes would also affect provider agents’ attitude toward the intervention. Hospital intervention and operating costs, both affecting hospital agents’ profit, were the other two sensitive parameters. Because the hospital agent has the financial capability and covered the main cost of the intervention (such as hiring care managers), SSP was not sensitive to the parameters on the PCP agent’s side.

## Discussion

How to provide incentives to improve shared accountability in U.S. healthcare systems is a difficult policy issue with a great deal of uncertainties and trade-offs [50]. The CHF agent-based simulation model demonstrated and quantified how different implementations of the ACO and the shared saving payment model can influence provider behavior regarding the evidence-based intervention and therefore generated different financial and quality outcomes. The model can help decision-makers better understand the complexity and risks of ACO design and facilitate more informed decision-making.

**Fig. 11:**
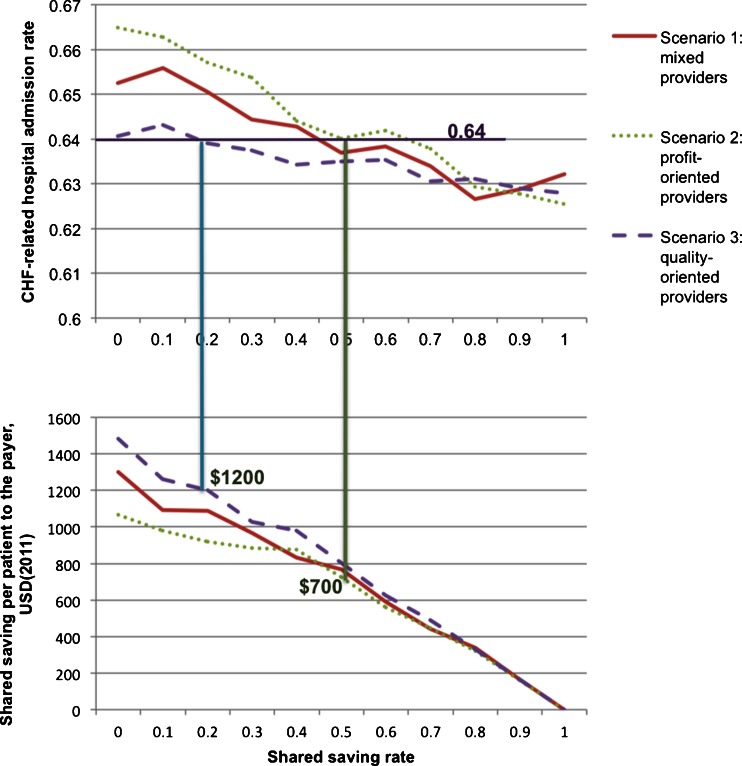
Effects of provider type on shared savings

From CMS’s perspective, objectives include both controlling the cost and promoting the quality of care. The scenario analysis showed that there was a trade-off between the payer’s financial return and the quality outcomes when setting SSR. The model can help decision-makers understand the constraints when determining SSR. In a hypothetical scenario, if CMS wants to achieve a quality target to control the CHF-related hospital admission rate to 64 % when all the providers are profit-oriented, the model results indicate that CMS should set SSR at 51 %, which would generate a $700 saving per CHF patient per year (shown in Fig. 11). The payer therefore would not achieve a financial objective, say $1,000 saving per patient, at the same time. Hence, with the understanding of these constraints, CMS can determine an SSR value that can balance its two objectives.

Once the SSR is decided, the next step is to determine an SRH value that can motivate both the hospital agents and PCP agents to work together on the CHF intervention. Creating a mechanism to promote cooperation between hospitals and physicians is difficult, and an immature payment model may incite tension and competition between hospitals and physicians [51]. The simulation results have identified different patterns of outcomes by SRH, which can help decision-makers choose the optimal SRH value to maximize the outcomes based on the configuration of SSR.

The model can help analyze how provider types and priorities could influence payment model designs. In the same hypothetical scenario shown in Fig. 11, if all the providers are quality-oriented, CMS can set SSR at 18 % to achieve the same goal of controlling the CHF-related hospital admission rate to 64 %. This setting will generate a $1,200 saving per CHF patient per year instead of a saving of $700 when all the providers are profit-oriented.

This result shows that the payer agent needs to provide different levels of incentives for different types of providers to achieve the same level of quality outcomes. This brings up the problem that the more profit-oriented a provider agent is, the more financial reward he/she will receive from the payer. This problem could be addressed by expanding the payer’s strategies to develop mixed motives to simulate all types of providers [52], such as aligning the payment with quality requirements, public reporting of quality performance, and initiating education programs to increase awareness of quality of care [53–55].

The sensitivity analysis showed that the outcomes were most sensitive to the effects of the intervention, followed by the intervention cost. Hence, selecting the cost-effective intervention is critical for the success of an ACO. For broader applications beyond CHF, ACO stakeholders need to wisely choose improvement opportunities to pursue based on their capability and current situations.

There are some limitations to the agent-based model for CHF care. First, the model parameters are based on the best information obtainable. The results reflect a national average trend for Medicare patients. Adjustments should be made when referring to some specific patient demographics, provider characteristics, and geographic locations. Second, the weight for each behavior-predicting factor in the provider decision module is not drawn from a survey of physicians. A national physician’s behavior survey with information to provide the values of these weights was not identified in a literature search. Hence, the users of the model can configure the provider agent parameters on the basis of the characteristics of the providers with whom they are working.

## Conclusion

The agent-based simulation model provides predictive analytics and recommendations for decision-makers to make informed decisions on how to design an ACO and its corresponding shared saving payment model, given their current provider and patient population environment, to maximize the desired outcomes.

The agent-based simulation model has identified critical determinants of an optimal design for the payment model that can motivate provider behavior changes to achieve maximum financial and quality outcomes. The model has quantified the trade-off between the payer’s financial return and the quality outcomes, helping decision-makers configure the shared saving model based on their objectives.

The complexity and risks of the ACO model design generate a need for modeling and simulation tools that are flexible, capable, and user-adjustable to facilitate the design and implementation of an ACO model for different disease conditions, payment models, and provider and patient agent characteristics. For future use of the model, users can, based on their situations and objectives, configure the ACO design parameters, generate corresponding patient and provider agents, and then analyze different scenarios and optimize their ACO models.

This study presents the promising application possibilities of ABM, an increasingly important method to support policy and management decision-making in broad and complex healthcare systems. Different from aggregated-level modeling methods, ABM can incorporate various goals and behaviors of individual stakeholders. It is also capable of modeling agent communication and interactions. These advantages make ABM a closer approximation of the real world. Also, because each agent represents a real entity in the health system (such as a physician or a patient), healthcare practitioners are likely to understand and accept the agent-based model. Therefore, the solutions recommended and insights generated for an ACO model design by the agent-based model increase the chance of improving the real health system.
